# Nanoscale enhancement of photoconductivity by localized charge traps in the grain structures of monolayer MoS_**2**_

**DOI:** 10.1038/s41598-018-34209-w

**Published:** 2018-10-25

**Authors:** Myungjae Yang, Tae-Young Kim, Takhee Lee, Seunghun Hong

**Affiliations:** Department of Physics and Astronomy, and Institute of Applied Physics, Seoul National University, Seoul, 08826 Korea

## Abstract

We report a method for mapping the nanoscale anomalous enhancement of photoconductivity by localized charge traps in the grain structures of a molybdenum disulfide (MoS_2_) monolayer. In this work, a monolayer MoS_2_ film was laterally scanned by a nanoscale conducting probe that was used to make direct contact with the MoS_2_ surface. Electrical currents and noise maps were measured through the probe. By analyzing the data, we obtained maps for the sheet resistance and charge trap density for the MoS_2_ grain structures. The maps clearly show grains for which sheet resistance and charge trap density were lower than those of the grain boundaries. Interestingly, we found an unusual inverse proportionality between the sheet resistance and charge trap density in the grains, which originated from the unique role of sulfur vacancies acting as both charge hopping sites and traps in monolayer MoS_2_. In addition, under light illumination, the larger the trap density of a region was, the larger the photocurrent of the region was, indicating anomalous enhancement of the photocurrent by traps. Since our method provides valuable insights to understand the nanoscale effects of traps on photoconductive charge transport, it can be a powerful tool for noise studies and the practical application of two-dimensional materials.

## Introduction

Atomically layered transition metal dichalcogenides (TMDCs) have emerged as promising two-dimensional materials for future applications^1,2^. Since TMDCs have unique properties, including an intrinsic band gap varying with the number of layers, a direct band gap and strong spin-orbit coupling in monolayers^2–4^, they have been widely studied^1–7^. As a typical TMDC material, molybdenum disulfide (MoS_2_) has a layered structure with a monolayer thickness of ~0.7 nm^1^. The layers of MoS_2_ are held together by weak van der Waals interactions^1,4^. Due to the direct band gap of ~1.8 eV in the monolayer^2^, MoS_2_ has great potential for device applications, especially in optoelectronics such as photodetectors^5^, light-emitting diodes^6^ and solar cells^7^. However, the nanoscale characteristics of electrical conduction and photoconduction in the grain structure of a MoS_2_ layer are not fully understood.

In many applications, electrical noise is an important factor, which significantly affects the performance of devices^8^. In addition, noise data often provide critical information for understanding the internal structures and defects of electronic materials such as MoS_2_, and thus, methods for the measurement of the noise source activities in electronic materials would be very useful tools for engineering high performance devices for optoelectronic^9^ and electrochemical applications^10^. Until now, noise studies for specific materials have usually been carried out using noise measurements on devices based on the materials. For example, the characteristics and origins of electrical noise in MoS_2_ devices were revealed by measuring the gating effect on electrical noise in MoS_2_-based electrical channels^11–14^. Meanwhile, a method using a conducting atomic force microscopy (AFM) enabled the direct imaging of localized noise sources such as charge traps in materials^15–17^. A previous study utilized this noise microscopy method to obtain a map of the charge traps on a graphene sample^15^. However, noise microscopy analysis of two-dimensional charge transport in a photoconductive channel has not been reported before.

Herein, we report the observation of the nanoscale anomalous enhancement of photoconductivity induced by localized charge traps in the grain structures of monolayer MoS_2_. In this work, a nanoscale conducting probe was used to make direct contact on a monolayer MoS_2_ sample on a SiO_2_ substrate, and, then, scanned laterally while mapping the electrical currents and noise through the probe. Then, the measured current and noise maps were analyzed to obtain maps for the sheet resistance and localized charge trap distributions in the MoS_2_ grain structures. The maps clearly show multiple MoS_2_ grains with rather low sheet resistances and charge trap densities compared with their boundaries. Interestingly, unlike other common conducting channels, the sheet resistance inside the grains was found to be inversely proportional to the charge trap density, which was attributed to the unique role of sulfur vacancies working as both charge hopping sites and charge traps in MoS_2_. Furthermore, under light illumination, regions with larger charge trap densities exhibited larger photocurrents, indicating that photocurrents were enhanced by charge trap sites. This method provides a valuable insight for the nanoscale effects of charge traps on the photoconductive charge transport, and, thus, can be utilized for various electrical noise research and practical device applications based on two-dimensional materials.

## Results and Discussion

### Experimental setup

**Figure 1:**
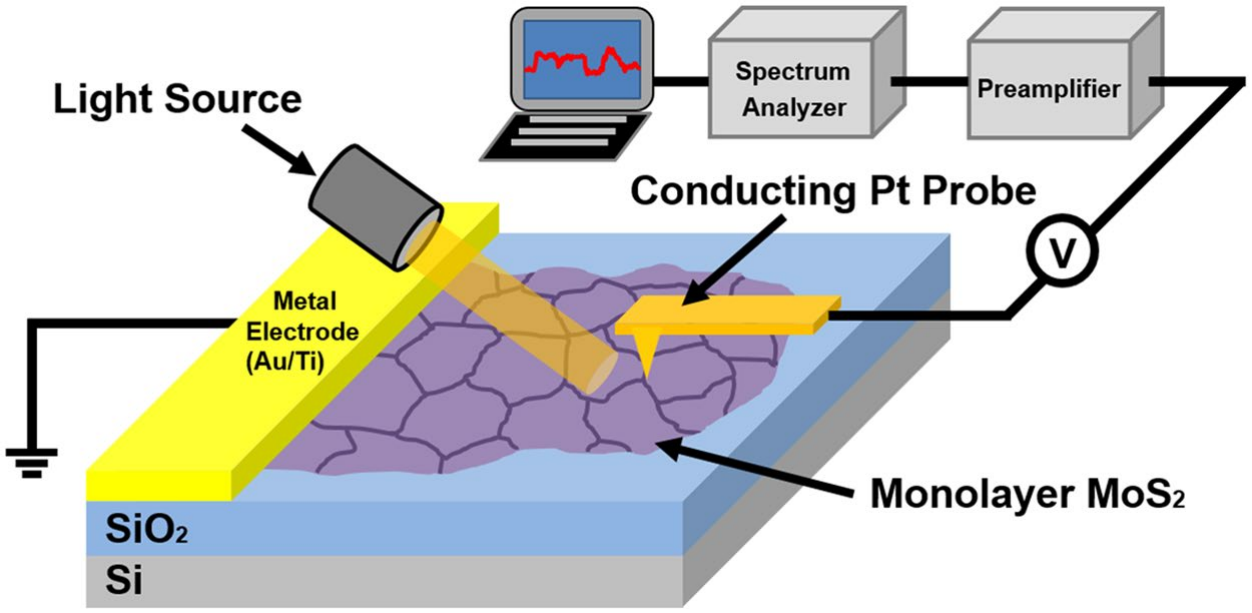
Schematic diagram depicting the scanning noise measurement setup for a MoS_2_ monolayer sample grown onto a SiO_2_/Si substrate. A conducting AFM probe laterally scanned the MoS_2_ surface under ambient conditions, while making direct contact with the sample. Electrical current and noise PSD maps were simultaneously measured through the probe. The measured data were analyzed to obtain maps of the sheet resistance and charge trap density distributions in MoS_2_ grain structures.

Figure 1 shows the schematic diagram of our current and noise measurement setup. A MoS_2_ monolayer film was grown onto a SiO_2_/Si substrate by a chemical vapor deposition (CVD) method. For the noise microscopy measurement, a Pt-based conducting probe (25Pt300B, Park Systems) installed in an AFM (XE-70, Park Systems) was used to make a direct contact with the surface of the MoS_2_ film under ambient conditions. Then, a direct current (DC) bias voltage of 5 V was applied to the Pt probe using a DC power supply (DS345, Stanford Research Systems). The current through the Pt probe was measured and converted to amplified voltage signals by a low-noise preamplifier (SR570, Stanford Research Systems). Simultaneously, electrical noise (the fluctuating component of a current signal) was collected using a band-pass filter (6 dB) in the SR570 preamplifier. We utilized a homemade root mean square (RMS)-to-DC converter to obtain the RMS power of the noise. The absolute noise power spectral density (PSD) at the central frequency of the band-pass filter was obtained by dividing the square of the measured RMS noise power with the bandwidth of the band-pass filter. Using this setup, two-dimensional maps for the topography, current and noise PSD were obtained at the same time by scanning the AFM probe on the MoS_2_ sample. The mapping data were analyzed to obtain maps of the sheet resistance and charge trap density. In addition, we measured changes in the current and noise maps by white light illumination using a light source (LS-F100HS).

### Characterization of CVD-grown monolayer MoS_2_

**Figure 2:**
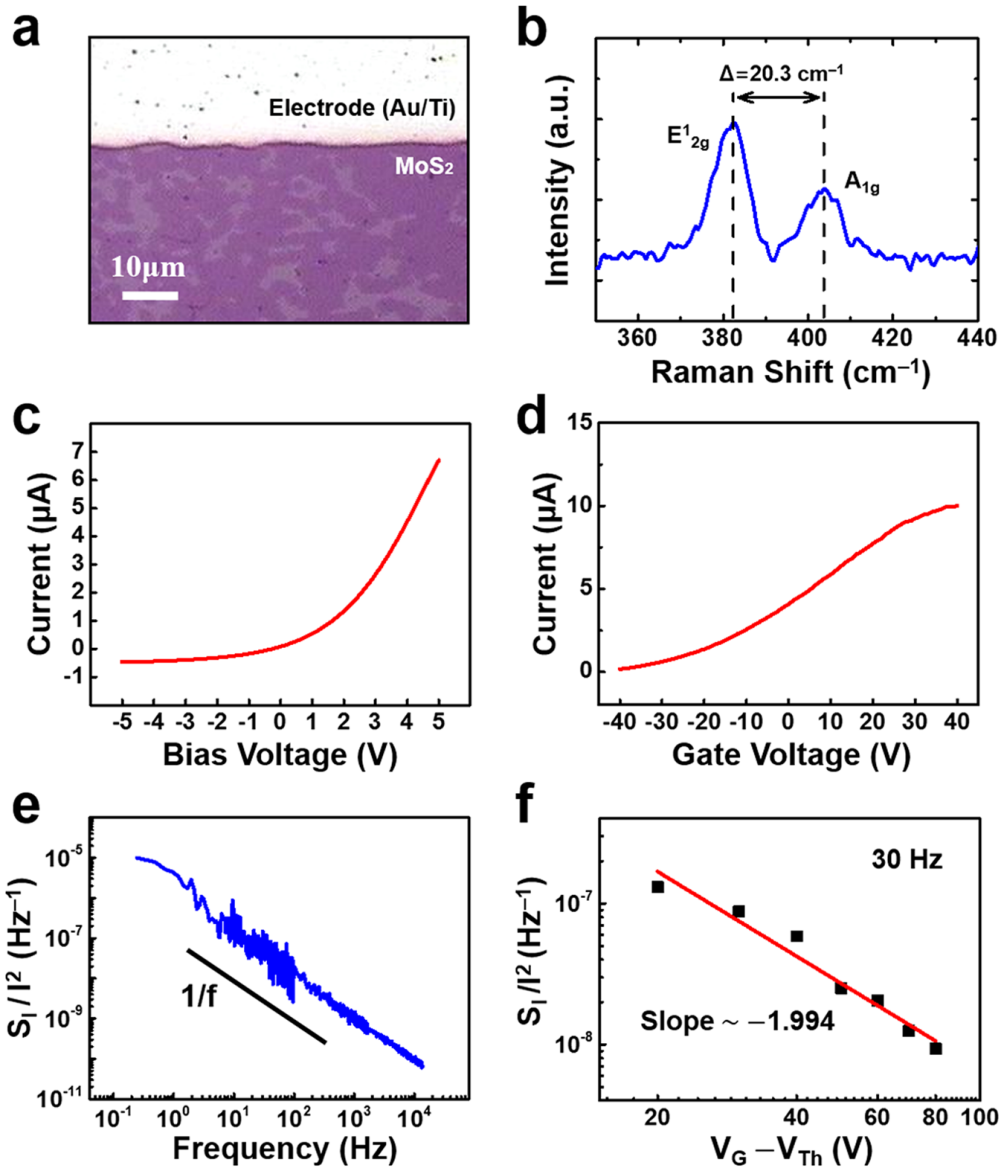
Characterization of a monolayer MoS_2_ sample. (**a**) Optical image of monolayer MoS_2_ grown onto a SiO_2_/Si substrate. The constant color contrast indicates the uniform thickness of the MoS_2_ film. (**b**) Raman spectroscopy data for the sample. Two major peaks at *383.4* and *403.7* cm^−1^ correspond to the *E*^1^_*2g*_ and *A*_*1g*_ modes of MoS_2_, respectively. The interval of 20.3 cm^−1^ between the two peaks indicates the monolayer of MoS_2_. (**c**) Current-voltage (*I-V*) curve measured on the sample. (**d**) Current measured by sweeping a gate voltage (*V*_G_). The threshold voltage (*V*_Th_) was estimated to be ~−40 V. (**e**) Current-normalized noise PSD (*S*_I_/*I*^2^) dependence on frequency. A 1/*f* noise behavior was observed. (**f**) *S*_I_/*I*^2^ at 30 Hz as a function of *V*_G_. The slope of the fitted line is −1.994, which is close to the value of −2 expected for carrier number fluctuations as the dominant origin for the noise generation.

Figure 2(a) shows the optical microscopy image of a MoS_2_ film. The MoS_2_ was synthesized via CVD on a SiO_2_/Si substrate. The MoS_2_ film exhibited a darker purple color than the SiO_2_ region. The constant color contrast of the MoS_2_ region implies that the thickness of the MoS_2_ film was rather uniform^18^. The image indicates that the surface of the MoS_2_ sample was uniform and clean.

The layer number of the MoS_2_ film was characterized by Raman spectroscopy (Fig. 2(b)). We used a Raman microscope (XperRam 200, Nanobase) with a 532 nm laser. The Raman spectrum of our MoS_2_ film shows two major peaks at 383.4 and 403.7 cm^−1^. The interval for the peaks is approximately 20.3 cm^−1^. These two peaks correspond to the E^1^_2g_ (383.4 cm^−1^) and A_1g_ (403.7 cm^−1^) modes of MoS_2_^18^. It is known that the interval for the two major peaks decreases as the number of layers in MoS_2_ decreases, being ~20 cm^−1^ in the case of a monolayer^19^. Hence, the Raman spectrum indicates that the MoS_2_ film consisted of a single atomic layer. The topography image of the MoS_2_ film obtained by AFM measurement also supported the observation of monolayer formation in our film (Fig. S1 in the Supplementary Information).

Figure 2(c) shows the current-voltage (*I*-*V*) characteristics of our MoS_2_ sample. A bias voltage was applied to the Pt probe and swept from −5 to 5 V. The *I*-*V* graph exhibits asymmetric and nonlinear behavior. The nonlinear curve implies that Schottky contacts are formed between the MoS_2_ layer and the electrode, presumably due to the large band gap of MoS_2_^20^. Since the work function of the Pt probe is larger than that of the Au/Ti electrode, a negative bias on the Pt probe would worsen the Schottky barrier between the MoS_2_ film and the Au/Ti electrode, resulting in a low current level. This result shows the Schottky barrier at the MoS_2_-metal contacts may have a significant effect on the *I*-*V* characteristics, as reported previously^21^. Figure 2(d) shows a back gate effect on the sample at a source-drain bias voltage of 3 V. The gate voltage (*V*_G_) was swept from −40 to 40 V using the SiO_2_ substrate as a back gate. The result shows an increasing current as the gate voltage was swept from negative to positive values, indicating typical *n*-type behavior for the MoS_2_ channel^1,11–14^. The threshold voltage (*V*_Th_) (the minimum gate voltage required to form a conducting channel) was obtained from the curve. The threshold voltage (*V*_Th_) was estimated to be ~−40 V, which is the *V*_G_-axis intercept of the extrapolated line for the maximum slope region in the curve. The electrical properties of the MoS_2_ sample were comparable to those found in a previous study^18^, confirming the uniform quality of our MoS_2_ film.

In Fig. 2(e), the frequency dependence of the current-normalized noise PSD (*S*_I_/*I*^2^) is plotted on a log scale. A spectrum analyzer (SR780, Stanford Research Systems) was utilized to measure the noise spectrum. The *S*_I_/*I*^2^ exhibited a 1/*f* noise behavior, as reported in previous noise studies on MoS_2_ devices^11–14^. In our previous work, we showed that noise PSDs exhibited 1/*f*^2^ (or Lorentzian) noise behavior when a current noise was generated by a few trap states that had rather uniform trapping times^15,17^. However, noise spectra exhibited 1/*f* behavior^15–17^ when there were many trap states with various trapping times. The 1/*f* noise behavior in our plot implies that the noise was generated by many different noise sources such as charge traps in the MoS_2_ sample.

Previously, a 1/*f* noise was suggested to originate from *mobility fluctuations* or *carrier number fluctuations* and that in each case *S*_I_/*I*^2^ is differently related to carrier density^10,11,22,23^. To clarify a dominant origin, we measured *S*_I_/*I*^2^ at different gate voltages (*V*_G_) since the carrier density can be modulated by *V*_G_. Fig. 2(f) shows a plot of *S*_I_/*I*^2^ values over *V*_G_. The *S*_I_/*I*^2^ was measured while a *V*_G_ ranging from −20 to 40 V was applied to the SiO_2_ back gate. The slope of the fitted line in the log-log plot is estimated to be −1.994, which is close to −2, as expected for *carrier number fluctuations*^14,22,23^. This implies that *carrier number fluctuations* were the dominant mechanism for noise generation in our MoS_2_ sample, as reported previously^12,14^.

### Charge trap distribution in the grain structure of monolayer MoS_2_

**Figure 3:**
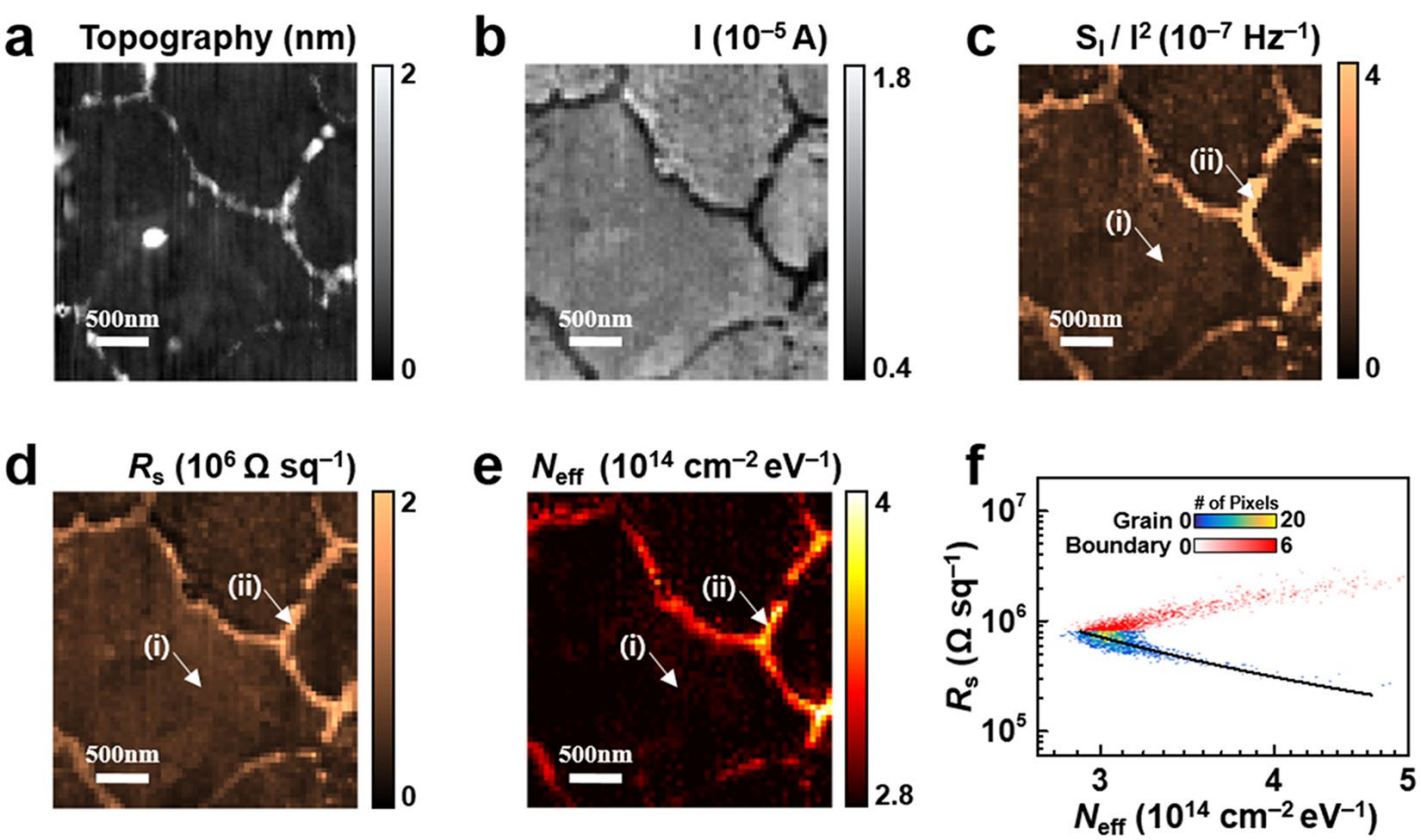
Mapping of sheet resistance and charge trap density in the grain structure of a MoS_2_ monolayer. (**a**) AFM topography image of the MoS_2_ monolayer film. (**b**) Current (*I*) map of the MoS_2_ film. A bias voltage of 5 V was applied to the film through a metal electrode. The data show clear separation between grains and boundaries. (**c**) Current-normalized noise PSD (*S*_I_/*I*^2^) map (at 17.3 Hz) for the film. The large *S*_I_/*I*^2^ values in the boundaries indicate that the existence of dense defects act as noise sources. (**d**) Sheet resistance (*R*_s_) map for the MoS_2_ sample. The boundary showed larger *R*_s_ than the grain due to structural disorder. (**e**) Charge trap density (*N*_eff_) map for the sample. The boundary regions showed higher *N*_eff_ than the grain regions. (**f**) Scatter plot showing the relation between *N*_eff_ and *R*_s_. A negative correlation between *N*_eff_ and *R*_s_ was observed in the grains, while the boundaries showed a positive correlation.

Figure 3(a) shows the AFM topography image of a MoS_2_ monolayer. The surface area (3 × 3 μm^2^) was scanned by an AFM probe. In the image, dark regions (film thickness <1 nm) are distinguished from bright regions (film thickness ~2 nm). Additionally, there is a bright dot near the center of the grain. From the reported thickness of a MoS_2_ monolayer (~0.7 nm)^1^, we can consider the dark area as grains of MoS_2_ and the bright area as grain boundaries. The dot near the center of the grain would be a precursor for the CVD process of a MoS_2_ film, as reported previously^24^. The difference in thickness between grain and boundary regions can be explained via a boundary formation mechanism. Typically, the growth of individual grains is known to stop when chemical bonds are formed between the grains^25^. However, it is common that the neighboring grains proceed to grow even after they encounter each other, forming additional layers. Thus, layer-overlapping without chemical bonds can occur, resulting in thicker boundaries^25^. The topography image indicates the cleanliness of the film, which showed no wrinkles or severely rough surface regions.

Figure 3(b) shows a current map for the MoS_2_ sample. The current was measured through the conducting probe while a DC bias voltage of 5 V was applied to the Au/Ti electrode. During the measurement, the sample was held under dark conditions to exclude photocurrents. In the map, bright and dark regions are clearly distinguished. The dark regions (~10^−6^ A), which correspond to boundary regions, showed lower currents compared to the grain regions (~10^−5^ A). This current difference could arise from structural disorder in the boundaries, which obstructs current flow. In a grain boundary region, the atomic structure could be different with that inside the grain due to the different orientation of adjacent grains, or grain overlapping and ruptures. Previous works show that various defects in boundaries, such as line dislocations and complex atomic ring structures, induce mid-gap states and decrease the band gap^26–29^. In addition, grain overlapping and ruptures could generate grain edges with unsaturated bonds, which lead to the intrinsic modification of the electronic structure due to the loss of periodicity^28^. The result shows success mapping of localized currents via a stable contact between the probe and the MoS_2_ film, which is important for reliable electrical measurements.

Figure 3(c) shows a *S*_I_/*I*^2^ map (at 17.3 Hz) obtained from a noise PSD map measured simultaneously with the current map (Fig. 3(b)). Here, the noise PSD map (*S*_I_) at 17.3 Hz was divided by the square of the current (*I*) map. The *S*_I_/*I*^2^ values were ~1.12 × 10^−7^ Hz^−1^ inside a grain (arrowed by (i)) and ~3.96 × 10^−7^ Hz^−1^ inside a boundary (arrowed by (ii)), indicating a higher noise level in the boundary than in the grain. The high *S*_I_/*I*^2^ in the boundary implies that a large electrical noise was generated in the grain boundaries. Previously, it was reported that defects and disorders in a MoS_2_ layer induce localized states within a band gap^30^, which can act as traps^31,32^ and generate current noise by trapping and detrapping charge carriers. Hence, the large electrical noise from boundaries can be attributed to rather high density of defects in the area. It is remarkable that noise contributions from each localized area for the monolayer MoS_2_ could be distinguished in our result, providing important information about how noise levels differ in grain structures.

Figure 3(d) shows a sheet resistance (*R*_s_) map of the monolayer MoS_2_ film. To obtain the *R*_s_ distribution, we performed computer calculations based on an iterative method developed in our previous work^15^. In brief, we calculated the *R*_s_ map, which reproduced the measured current map in Fig. 3(b), via an iterative method. In the map, grains and their boundaries exhibit a clear difference in *R*_s_. The *R*_s_ values were ~8.28 × 10^5^ Ω sq^−1^ inside a grain (arrowed by (i)) and ~2.05 × 10^6^ Ω sq^−1^ inside a boundary (arrowed by (ii)), showing ~2.5 times higher values in boundaries than in grains. The result was consistent with the reported *R*_s_ values for CVD-grown monolayer MoS_2_ (10^5^–10^6^ Ω sq^−1^)^6,26,33^. The large *R*_s_ of the boundary regions could originate from numerous scattering centers due to structural disorder^25,34^. Additionally, the *R*_s_ difference between grains and boundaries was similar to that measured for intra- and inter-grain channels in field effect transistors (FETs) in a previous study^26^. However, it should be mentioned that previous measurements had difficulty in clearly distinguishing the localized resistance values for the grains and boundaries since the FET channels included both regions.

From the data in Fig. 2(f), we showed that the 1/*f* noise of our MoS_2_ channel (Fig. 2(e)) was mainly generated by carrier number fluctuations. The number of carriers fluctuates since charge carriers are randomly trapped and detrapped by charge trap states, generating current noise^35^. Using the differential method developed in our previous study^15^, an effective charge trap density (*N*_eff_) (the integrated value of the charge trap density over the thickness) of a small area Δ*x*Δ*y* at (*x, y*) on a sample surface is obtained as

1 $${N}_{{\rm{eff}}}(f,x,y)=\frac{{({\rm{\Delta }}C)}^{2}}{{(I)}^{2}}\frac{f}{kT}\times \frac{{\rm{\Delta }}{S}_{{\rm{I}}}(f,x,y)}{{\rm{\Delta }}x{\rm{\Delta }}y}$$

where Δ*C*, *I*, *f*, *k*, *T* and Δ*S*_I_ are the number of charge carriers, measured current, frequency, Boltzmann constant, temperature and noise PSD generated by the small area, respectively. In the case of 1/*f* noise, Δ*S*_I_(*f, x, y*) is proportional to 1/*f*. Then, *f* Δ*S*_I_ becomes *f*-independent, resulting in a *f*-independent *N*_eff_(*f*, *x*, *y*), i.e., *N*_eff_(*x*, *y*). To estimate Δ*S*_I_, we considered a MoS_2_ layer as a two-dimensional resistance network. This “network model” was shown to be a successful model in our previous study on graphene samples^15^. The Δ*C* in equation (1) was estimated from the charge carrier concentration. We calculated the charge carrier concentration (*n*) using *n* = *C*_OX_ (*V*_G_ − *V*_Th_)/*e*, where *C*_OX_ is the gate capacitance of the SiO_2_ layer (~1.48 × 10^−8^ F/cm^2^), *V*_G_ is the gate voltage (0 V), *V*_Th_ is the threshold voltage (~−40 V), and *e* is the elementary charge (1.60 × 10^−19^ C). The calculated value for *n* was ~3.69 × 10^12^ cm^−2^. Then, Δ*C* could be calculated from Δ*C* = *n*Δ*x*Δ*y*, where Δ*x*Δ*y* is the effective contact area of the conducting Pt probe (~2000 nm^2^). Eventually, we could estimate *N*_eff_ values at each point of the area scanned by a conducting AFM probe. Since *N*_eff_ is an integrated value over a thickness, it will be a useful value representing the effective density of charge traps in two-dimensional materials.

Figure 3(e) shows the effective charge trap density (*N*_eff_) map of the MoS_2_ monolayer. The map exhibits the areal density distribution of the charge traps on the sample. The *N*_eff_ value was ~2.91 × 10^14^ cm^−2^ eV^−1^ inside a grain (arrowed by (i)), while the boundary region (arrowed by (ii)) exhibited a *N*_eff_ value of ~3.87 × 10^14^ cm^−2^ eV^−1^, which was ~1.3 times higher in value than that of the grain. In a MoS_2_ film, charge traps can be induced by defects including atomic vacancies, dangling bonds and impurities^30–32^. Since defects in monolayer MoS_2_ generate trap states within a band gap while defect-free monolayer MoS_2_ shows no such states^30,36^, *N*_eff_ corresponds to the density of traps generated by defects. The high *N*_eff_ in the boundary implies the existence of abundant charge traps, which originate from structural disorder of the boundaries^25,34^. Charge traps in a MoS_2_ sample can also be located in the underlying substrate. However, it should be noted that the *N*_eff_ values in our map were 2-3 orders of magnitude higher than the reported oxide trap density associated with SiO_2_ substrates^12,37^, indicating that the substrate was not the main origin for the generated noise. Instead, the *N*_eff_ could be attributed to defects generated during the CVD process under high temperature (~750 °C) and low pressure (~10 Torr), as reported previously^38^. Previously, it was reported that the presence of defects such as sulfur vacancies significantly affects the band structure of monolayer MoS_2_ by introducing localized mid-gap states near the Fermi level, leading to a transition from a direct to indirect band gap^30^. Our method provides a method to map localized density of charge traps in two-dimensional nanomaterials, which can be useful for studying charge traps in various other nanostructured materials.

Figure 3(f) shows a scatter plot for the relationship between *N*_eff_ and *R*_s_ for monolayer MoS_2_ on a log-log scale. Each data point in the plot is obtained from the pixel area in the *N*_eff_ map (Fig. 3(e)) and the corresponding pixel area in the *R*_s_ map (Fig. 3(d)). The data points for the grains and boundaries were distinguished using the *R*_s_ map. The plot exhibits different tendencies for grain and boundary regions, showing a positive correlation in boundaries and a negative correlation in grains. The positive *N*_eff_-*R*_s_ correlation in boundaries is similar to previously reported results on the relation between noise PSD and resistance in percolated systems^38–41^. Since boundaries are highly disordered regions with many trap states, each pixel region in the boundaries can be considered as a localized percolation channel. In such a system, it was reported that both resistance and its fluctuation have a power law dependence on (*p* − *p*_*c*_), where *p* is the fraction of the conductive paths and *p*_*c*_ is the percolation threshold^39^. Hence, the resistance of each pixel region (*R*_s_, in our case) is related to its fluctuation as follows

2 $$\frac{{\rm{\Delta }}{S}_{{\rm{R}}}}{{({R}_{s})}^{2}}\propto {({R}_{s})}^{w}\,\,(at\,p > {p}_{{\rm{c}}})$$

where Δ*S*_R_ is the PSD of *R*_s_. From *N*_eff_ ~ (Δ*S*_I_/*I*^2^) = (Δ*S*_R_/*R*_s_^2^), the relation *N*_eff_ ~ (*R*_s_)^*w*^ is obtained, which explains the correlation between *N*_eff_ and *R*_s_. In our result, the exponent *w* was approximately 0.4, which is similar to that observed in an FET channel including the grain boundary in monolayer MoS_2_^40^. This kind of scaling behavior has been observed in many percolation systems where the exponents varied according to the material, geometry and temperature^38–41^. On the other hand, the unique negative correlation for the grain regions can be explained by the electrical transport mechanism in monolayer MoS_2_. It was suggested that the charge transport in few-layered MoS_2_ is dominated by hopping through localized states, which originates from sulfur vacancies^14,30,42^. At room temperature (~300 K), the dominating transport mechanism in MoS_2_ should be nearest-neighbor hopping^30^. In this mechanism, the conductivity (*σ*) is expressed as *σ* ~ exp (−1/*a*^2^*kTN*_μ_), where ‘*a*’ is an average defect distance and *N*_μ_ is the density of states near the Fermi level, which corresponds to *N*_eff_^30^. The equation indicates that if there are many sulfur vacancies in a region (high *N*_eff_), the hopping probability for the carriers will be high due to the high density of vacancy-induced hopping sites, resulting in a high conductivity. In the plot, the data points for the grain regions are fitted well by the equation *R*_s_ ~ exp (1/*a*^2^*kTN*_eff_). As a fitting parameter, the average defect distance (*a*) was estimated to be ~1.97 nm, which was very close to the reported value of ~1.7 nm^30^. Previous observations based on transmission electron microscopy (TEM) revealed that sulfur vacancies are the most common defects in monolayer MoS_2_^30,36^. Additionally, it should be mentioned that the reported density of trap states from sulfur vacancies was comparable to our *N*_eff_ value^30^. Therefore, the negative correlation between *N*_eff_ and *R*_s_ for the grain regions could be attributed to the sulfur vacancies playing a key role for both *charge transport* and *charge trap generation* in monolayer MoS_2_.

### Enhancement of photoconductivity by localized charge traps

**Figure 4:**
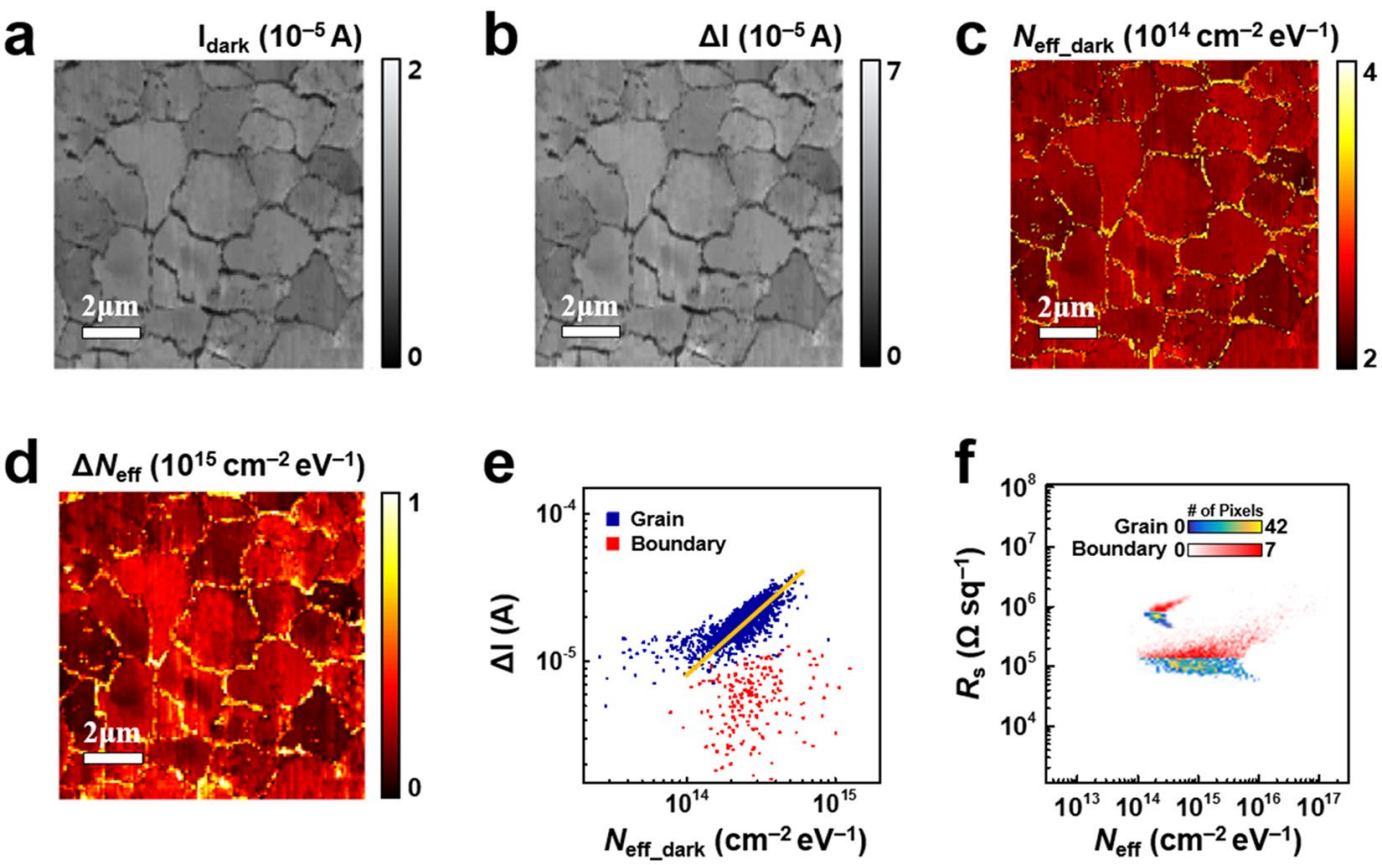
Changes in the current and charge trap density of a MoS_2_ monolayer due to light illumination. (**a**) Current map measured under dark conditions. (**b**) Map of current change (Δ*I*) under illumination. Photocurrents were larger in grains than in boundaries. (**c**) Map of the charge trap density under the dark condition (*N*_eff_dark_). (**d**) Map showing changes in charge trap density (Δ*N*_eff_) due to illumination. The positive Δ*N*_eff_ arises from defect generation by absorbed photons. (**e**) Scatter plot exhibiting a correlation between *N*_eff_dark_ and Δ*I*. The slope of the orange fitted line is ~0.9, indicating linear proportionality. (**f**) Scatter plot of *N*_eff_ *versus R*_s_ under dark and illuminated conditions. The *R*_s_ generally decreased while the distribution of *N*_eff_ was broadened in both grains and boundaries.

To investigate the effect of charge traps on photoconductive charge transport in monolayer MoS_2_, we measured the MoS_2_ film under dark and illuminated conditions. Figure 4(a) shows a current map (*I*_dark_) of the MoS_2_ monolayer measured under the dark condition. A DC bias voltage of 5 V was applied to the Au/Ti electrode. In the map, many grains were observed since we scanned an area (10 × 10 μm^2^) larger than that of individual grains. The *grains* and *boundaries* were distinguished by relatively *high* and *low* current values, respectively.

Figure 4(b) is the map of the current changes (Δ*I*) caused by light illumination. We used a white light source (LS-F100HS, Light Bank) with an intensity of ~100 mW/cm^2^. Here, a current map was first measured under the dark condition, with the measurement repeated under illumination. After illumination, the currents in the grains were found to increase by more than 10^−5^ A, resulting in ~7.5 times higher current values than before illumination. On the other hand, the currents in the boundaries increased by only a factor of ~4, indicating rather small changes (10^−6^-10^−5^ A). The current increase was caused by photocarrier generation in the MoS_2_ layer^9,43^. Previously, defects such as charge traps in MoS_2_ were reported to assist the recombination of photogenerated carriers as recombination centers, and, thus, to reduce the photocurrents^43^, which explains the low photocurrent in boundaries. It is also notable that there were clear differences in current values between individual grains. The current differences between individual grains were as large as 30% of the average current value of all the grains. This inter-grain current difference can be attributed to an energy band modulation that presumably arises from the random crystal orientation and strain of MoS_2_ grains grown via the CVD method^24,34^.

Figure 4(c) shows a map of the charge trap density when the MoS_2_ film was under the dark condition (*N*_eff_dark_). The *N*_eff_dark_ map was calculated using the current map (Fig. 4(a)) and the noise PSD map at 17.3 Hz. The *N*_eff_dark_ values were on the order ~10^14^ cm^−2^ eV^−1^ and found to be higher in boundaries than in grains. This result is consistent with that shown in Fig. 3(e). The difference in *N*_eff_dark_ between individual grains could arise from the grain-growing process, as mentioned in Fig. 4(b).

Figure 4(d) shows a map of the charge trap density changes (Δ*N*_eff_) in the MoS_2_ film due to illumination. The map was obtained by subtracting the *N*_eff_dark_ map (Fig. 4(c)) from a *N*_eff_ map measured under light illumination. The Δ*N*_eff_ map shows a considerable increase in the number of charge traps. The average values for Δ*N*_eff_ were ~2.85 × 10^14^ cm^−2^ eV^−1^ in grains and ~7.03 × 10^14^ cm^−2^ eV^−1^ in boundaries. This result was in accordance with previous studies showing that light irradiation can increase the charge traps in MoS_2_ layers by various mechanisms such as bond breaking, removal of atoms^44,45^. On the other hand, O_2_ or water molecules physisorbed onto MoS_2_ surfaces can work as a charge traps. Such physisorbed molecules could be removed by light illumination, reducing the charge trap density *N*_eff_. The large increase in charge trap density in our experiments indicates that charge trap generation by light illumination overwhelmed the effect of the desorption of such physisorbed molecular species.

Figure 4(e) shows a scatter plot showing the relation between the charge trap density under the dark condition *N*_eff_dark_ and photocurrent level Δ*I* in the grains and boundaries of the MoS_2_ film. Each data point in the plot represents *N*_eff_dark_ and Δ*I* values for the pixel area shown in Fig. 4b,c. In the plot, the data points for the boundary regions show no special correlation. However, a positive correlation between *N*_eff_dark_ and Δ*I* is observed in the grain regions, as indicated by the yellow fitted line with a slope of ~0.9. Considering that traps were previously reported to act as recombination centers, resulting in a decreased photocurrent^46^, this result is quite unusual. One possible explanation for this anomalous enhancement of the photocurrent by charge traps is that the photocurrent in few-layer MoS_2_ is significantly affected by O_2_ molecules on the MoS_2_ surface^43,47^. Previous studies showed that both dark current and photocurrent levels for monolayer MoS_2_ were much lower in ambient air than in vacuum, attributing the difference to chemisorbed O_2_ molecules, which captured a large portion of the electrons from MoS_2_^43,47^. When the MoS_2_ was illuminated with light under ambient conditions, electron-hole pairs were generated by photoexcitation. Then, the photogenerated holes recombined with the electrons captured by some chemisorbed O_2_ molecules, and the discharged O_2_ molecules were desorbed from the surface^43,47^. In this case, the photogenerated electrons, which might be captured by the chemisorbed O_2_ molecules, were now free and acted as a charge carrier. Thus, the number of free electrons in the MoS_2_ channel increased, and the current increase (Δ*I*) should be proportional to the number of desorbed O_2_ molecules generated by illumination. On the other hand, before illumination, the O_2_ molecules were chemisorbed on sulfur vacancies^9^ which were responsible for *N*_eff_dark_. In this case, the MoS_2_ regions with *more sulfur vacancies (or larger N*_eff_dark_) should have *more chemisorbed O*_2_ *molecules* before illumination^30,36^, and, following light illumination, they should also have a *larger number of desorbed O*_2_ *molecules* and *a larger current increase*. Therefore, the proportional relationships lead to the proportionality between Δ*I* and *N*_eff_dark_, explaining the fitted line with a slope close to 1 in the log-log plot. Our result revealed the anomalous enhancement of the photocurrent due to noise sources such as charge traps, which can be important in applications of MoS_2_ to optoelectronics since electrical noise is often a key parameter determining device performance.

Figure 4(f) shows a scatter plot showing the distributions of the *N*_eff_ and *R*_s_ values for the monolayer MoS_2_ under dark and illuminated conditions. Each data point represents the *N*_eff_ and *R*_s_ values of each pixel area in the maps. There are two groups of data points obtained under dark (high *R*_s_) and illuminated (low *R*_s_) conditions. Each group consists of the data points from grains and boundaries, which were distinguished using *R*_s_ maps (Fig. S2 in the Supplementary Information). The distribution of data points obtained under the dark condition was similar to that shown in Fig. 3(f), exhibiting different *N*_eff_-*R*_s_ correlations between grains and boundaries. On the other hand, under illumination, *R*_s_ was generally decreased while the distribution of *N*_eff_ was broadened in both grain and boundary regions. The broadening of the *N*_eff_ distribution was the result of a large increase in *N*_eff_ in most regions and a decrease in *N*_eff_ in only a few regions. The increase in *N*_eff_ was mainly caused by defect generation due to absorbed photon energy^44,45^. Additionally, localized states filled with charges under the dark condition could trap charge carriers after photoexcitation of the occupied charges, contributing to the increase in *N*_eff_. On the other hand, the desorption of physically adsorbed O_2_ molecules from the MoS_2_ surface could decrease *N*_eff_. It is also worth mentioning that the *N*_eff_ distribution is rather broadened by the light illumination, resulting in weakened *N*_eff_-*R*_s_ correlation. Presumably, under dark conditions, most of the charge traps are sulfur vacancies contributing to charge conduction as a hopping site^30^. However, light illumination generated various charge traps, which may not work as a hopping site, such as Mo vacancies and large atomic holes^44^.

## Conclusions

In conclusion, we successfully imaged how localized charge traps enhanced the photoconductivity in the grain structures of monolayer MoS_2_. By laterally scanning a conducting AFM probe used to make direct contact with a monolayer MoS_2_ sample, electrical currents and noise transmitted through the probe were simultaneously mapped. The mapping data were analyzed to obtain the distribution maps for the sheet resistance and charge trap density in the grain structures of MoS_2_. The result showed that both the sheet resistance and charge trap density were higher in the grain boundaries than in the grains. We found a unique negative correlation between the charge trap density and sheet resistance, which was attributed to the role of sulfur vacancies acting as both hopping sites and charge traps in monolayer MoS_2_. Furthermore, the photocurrent exhibited a positive scaling relation with the charge trap density since photogenerated holes recombined with electrons captured by oxygen molecules absorbed on sulfur vacancies. Since our strategy enabled us to map the nanoscale effect of localized charge traps on the photoconductive carrier transport, it should be a versatile tool that can be used for basic noise studies and applications based on versatile two-dimensional materials.

## Methods

### Monolayer MoS_2_ synthesis

Large-area monolayer MoS_2_ films were grown by a dual-heating zone chemical vapor deposition (CVD) system. The molybdenum trioxide (MoO_3_) powder and a carefully cleaned SiO_2_ substrate were heated inside a furnace up to ~750 °C, with the sulfur (S) powder heated inside an electric heater up to ~200 °C. The pressure inside a quartz tube was maintained at ~10 Torr with Ar carrying gas.

### Au/Ti contact electrode deposition

The CVD-grown MoS_2_ film was covered by a shadow mask used for electrode patterning without any surface treatments. To fabricate the electrodes, Ti (10 nm) and Au (100 nm) were deposited with a deposition rate of 0.5 Å/s at a pressure of ~10^−6^ Torr in an electron beam evaporator system.

### Raman spectroscopy

Raman spectra were measured for the CVD-grown MoS_2_ to characterize the thickness. The measurements were performed with a Raman spectroscopy system (XperRam 200, Nanobase) using a 532 nm laser.

## Electronic supplementary material

Supplementary Information
