# Supplementary material for: Nanoscale enhancement of photoconductivity by localized charge traps in the grain structures of monolayer MoS2
Source: Sci Rep. 2018 Oct 25;8:15822. doi: 10.1038/s41598-018-34209-w (PMC6202400; doi:10.1038/s41598-018-34209-w)
Supplement: Supplementary file 1 — Supplementary Information [file 41598_2018_34209_MOESM1_ESM.docx]

**[Supplementary Information]**

Nanoscale enhancement of photoconductivity by localized charge traps in the grain structures of monolayer MoS_2_

Myungjae Yang, Tae-Young Kim, Takhee Lee, and Seunghun Hong*

Department of Physics and Astronomy, and Institute of Applied Physics, Seoul National University, Seoul 08826, Korea

*E-mail: [seunghun@snu.ac.kr](mailto:seunghun@snu.ac.kr)


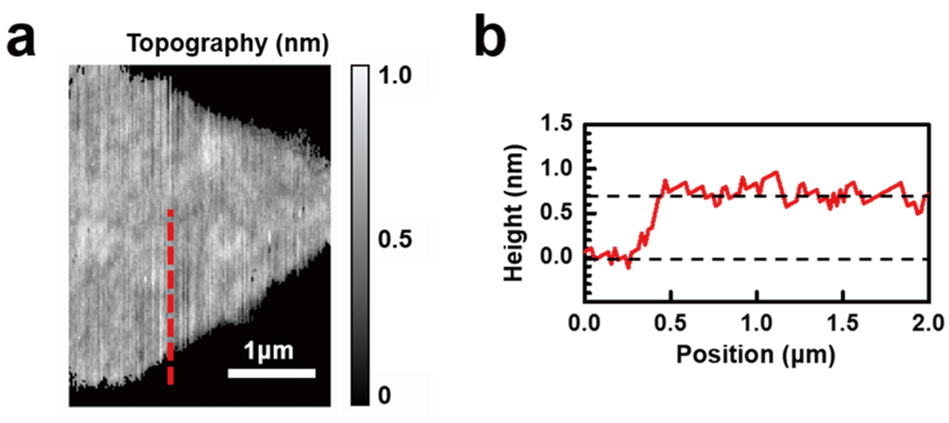


**Figure S1. Thickness profile of CVD-grown monolayer MoS_2_.** (a) AFM Topography image of a monolayer MoS_2_ sample. Dark regions are SiO_2_. (b) Height profile along a red dotted line in (a). The thickness was ~0.7 nm.


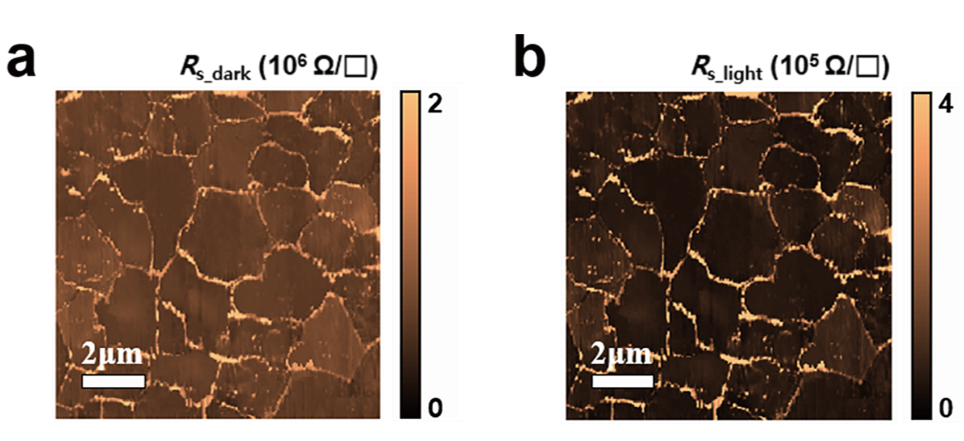


**Figure S2. Change in the sheet resistance by light illumination. S**heet resistance (*R*_s_) maps obtained under (a) dark and (b) illuminated conditions.
